# Transponder-Aided Joint Calibration and Synchronization Compensation for Distributed Radar Systems

**DOI:** 10.1371/journal.pone.0119174

**Published:** 2015-03-20

**Authors:** Wen-Qin Wang

**Affiliations:** School of Communication and Information Engineering, University of Electronic Science and Technology of China, Chengdu, P. R. China; University of Chinese Academy of Sciences, CHINA

## Abstract

High-precision radiometric calibration and synchronization compensation must be provided for distributed radar system due to separate transmitters and receivers. This paper proposes a transponder-aided joint radiometric calibration, motion compensation and synchronization for distributed radar remote sensing. As the transponder signal can be separated from the normal radar returns, it is used to calibrate the distributed radar for radiometry. Meanwhile, the distributed radar motion compensation and synchronization compensation algorithms are presented by utilizing the transponder signals. This method requires no hardware modifications to both the normal radar transmitter and receiver and no change to the operating pulse repetition frequency (PRF). The distributed radar radiometric calibration and synchronization compensation require only one transponder, but the motion compensation requires six transponders because there are six independent variables in the distributed radar geometry. Furthermore, a maximum likelihood method is used to estimate the transponder signal parameters. The proposed methods are verified by simulation results.

## Introduction

Distributed radar system operating with separated transmitters and receivers offers many operational advantages [1–4] to conventional monostatic and multi-frequency or multi-polarized radars [5–8], like the exploitation of additional information contained in bistatic reflectivity of targets [9], reduced vulnerability [10], and forward-looking imaging [11]. Distributed radar may offer reduced vulnerability to countermeasures such as jamming, as well as increased slow-moving target detection and identification capability via clutter tuning, in which the receiver maneuvers so that its motion compensates for the motion of the illuminator to create a zero Doppler shift for the area being searched. This could be worthwhile, e.g., for topographic features and drainage, to show the relationships that occur between forest, vegetation, and soils. This also provides important information for land classification and land-use management such as agriculture monitoring, soil mapping, and archaeological investigation. Attracted by these special advantages, various spaceborne and airborne distributed radar missions have been suggested or developed [12].

However, in a distributed radar the receiver uses an oscillator that is spatially displaced from that of the transmitter; hence, the phase noise of two independent oscillators cannot be canceled out. This superposed phase noise corrupts the received radar signal over the whole coherent integration time, and may significantly degrade subsequent imaging performance. Even when low-frequency or quadratic phase errors as large as 45 degree in a coherent processing interval can be tolerated, the requirement of frequency stability is only achieved by using ultra high-quality oscillators [13]. In the example of the bistatic spaceborne radar system TanDEM-X [2, 14], the relative phase has to be measured with at least 1 Hz sampling frequency in order to follow, unwrap and compensate the oscillator phase drifts within the acquisition [15, 16]. Furthermore, aggravating circumstances are often accompanied for airborne platforms due to different platform motions, the frequency stability will be further degraded. Thus, frequency synchronization compensation is required for distributed radar systems.

There is relative lack of practical synchronization technique for distributed radar systems. Since distributed radar is of great scientific and technological interest, several potential synchronization techniques have been suggested. The use of duplex links for oscillator frequency drift compensation was proposed in [14]. This concept is similar to the microwave ranging technique. However, this two-way operation is too complex to be applied for multistatic radar systems. We have investigated a direct-path signal-based phase synchronization technique in [17]. To receive the direct-path signal, the receiver must fly with a sufficient altitude and position to maintain line-of-sight contact with the transmitter/illuminator. In [18], we propose a time and phase synchronization method via global positioning systems (GPS) disciplined oscillators, but the GPS signals may be not available in some scenes. In this case, some other synchronization methods should be applied.

In terms of radiometry, the major goal in utilizing radar data is to infer some geophysical parameters about target areas within the scene via analysis of the recorded radar signal. The stability and consistency of the relation between the output voltage and antenna temperature, that is, the system gain is critical for quantitative remote sensing. Ideally, all radar imageries are absolutely calibrated such that the image pixel intensity is directly expressed in terms of the mean surface backscatter coefficients. This procedure, referred to as radiometric calibration [19], establishes a common basis for all image pixels, such that a given pixel intensity value represents an unique value of the backscattered signal power [20–22]. Internal calibration mechanisms indeed can be used to determine short term drifts, but an external calibration also is often required to provide a quantitative value for the measured backscatter and remove the system distortion. Although many calibration techniques have been developed [23–26], there are various disadvantages for passive calibrators due to their small radar cross-section. The most serious challenge is to find an area free of interference from man-made targets, namely, buildings and automobiles. It is well known that radar imagery is derived through correlating the raw data with a two-dimensional (2D) reference function. The azimuth component describes the phase history between the radar and target at a constant range, and the range component describes the phase history of the transmitted signal. An active calibrator provides the opportunity to change the phase history of the radar returns in either range or azimuth. Thus, modifying the retransmitted signal phase can displace the calibrator response in the final radar imagery. Additionally, it is useful to have an active calibrator that could shift its response location away from its physical location to a low backscatter area.

Literature search reveals that little work on distributed radar radiometric calibration has been published. But it is of great scientific and technological interest. A novel transponder used for calibrating high-resolution imaging radars was proposed in [27], which retransmits the original radar signal with two artificial Doppler shifts to the receiver. If the artificial Doppler shifts are chosen to be larger than the Doppler bandwidth of the raw data, then the transponder signal can be separated during subsequent radar signal processing. The details can be found in [27, 28]. In fact, the transponder can also be used for many other applications [29, 30].

This paper uses the transponder to jointly calibrate and synchronize airborne distributed radar for high-resolution remote sensing. Placing multiple transponders in both the along-track and cross-track dimensions, each transponder uses a distinct modulation frequency and yields an independent signal. Besides radiometric calibration, the motion and synchronization errors are also compensated by correlating the data collected from the transponder and distributed radar receivers. Moreover, a maximum likelihood (ML) algorithm is used to estimate the transponder signal parameters.

## Method

### Problem Formation

Because distributed radar is a coherent system, to complete coherent accumulation in azimuth, the radar echoes of equal range but different azimuth time should have the same phase after range compression and range migration correction. Since the frequency synchronization errors in bistatic radar system are caused mainly by the phase noise in the local oscillator (LO), the modulation waveform used for range resolution can be ignored and the distributed radar model can be simplified to an ‘‘azimuth only’’ system [31].

Suppose the transmitted signal is sinusoidal whose phase argument is

$$\begin{array}{c} \Phi_{T}\left(t\right)=2\pi f_{T}\left(t\right)t+\varphi_{T0}, \end{array}$$ (1)

where *φ*_*T*0_ is the transmitter original phase, *f*_*T*_(*t*) is transmitter carrier frequency which can be expressed as

$$\begin{array}{c} f_{T}\left(t\right)=f_{0}\left[1+\delta_{T}\left(t\right)\right], \end{array}$$ (2)

where *f*_0_ is the error-free carrier frequency and *δ*_*T*_(*t*) is the frequency fluctuation function. The receiver LO phase has the same form:

$$\begin{array}{c} \Phi_{R}\left(t\right)=2\pi f_{R}\left(t\right)t+\varphi_{R0}, \end{array}$$ (3)

where *φ*_*R*0_ is the frequency fluctuation and *f*_*R*_(*t*) is the receiver carrier frequency expressed as

$$\begin{array}{c} f_{R}\;\left(t\right)=f_{0}\left[1+\delta_{R}\;\left(t\right)\right]. \end{array}$$ (4)

Suppose the transmit time is *t*_0_, for a time delay *t*_*d*_ we can obtain the results by demodulating the received signal phase with the receiver LO phase

$$\begin{array}{c} \begin{array}{cc} \phi_{t0}= & \int_{0}^{t_{0}}2\pi \;\left[f_{R}\left(t\right)-f_{T}\left(t\right)\right]\;\text{d}t+\int_{t_{0}}^{t_{0}+t_{d}}2\pi f_{R}\left(t\right)\text{d}t+\varphi_{R0}-\varphi_{T0}\\ = & \varphi_{t0}+\varphi_{d0}+\varphi_{0}, \end{array} \end{array}$$ (5)

where

$$\begin{array}{c} \varphi_{t0}=\int_{0}^{t_{0}}2\pi \;\left[f_{R}\left(t\right)-f_{T}\left(t\right)\right]\;\text{d}t, \end{array}$$ (6a)

$$\begin{array}{c} \varphi_{d0}=\int_{t_{0}}^{t_{0}+t_{d}}2\pi f_{R}\left(t\right)\text{d}t, \end{array}$$ (6b)

$$\begin{array}{c} \varphi_{0}=\varphi_{R0}-\varphi_{T0}. \end{array}$$ (6c)

Analogously, suppose the transmit time is *t*_1_, we can get similar results

$$\begin{array}{c} \begin{array}{cc} \phi_{t1}= & \int_{0}^{t_{1}}2\pi \;\left[f_{R}\left(t\right)-f_{T}\left(t\right)\right]\;\text{d}t+\int_{t_{1}}^{t_{1}+t_{d}}2\pi f_{R}\left(t\right)\text{d}t+\varphi_{R0}-\varphi_{T0}\\ = & \varphi_{t1}+\varphi_{d1}+\varphi_{0}, \end{array} \end{array}$$ (7)

where

$$\begin{array}{c} \varphi_{t1}=\int_{0}^{t_{1}}2\pi \;\left[f_{R}\left(t\right)-f_{T}\left(t\right)\right]\;\text{d}t, \end{array}$$ (8a)

$$\begin{array}{c} \varphi_{d1}=\int_{t_{1}}^{t_{1}+t_{d}}2\pi f_{R}\left(t\right)\text{d}t. \end{array}$$ (8b)

From (5) and (7), we can express the phase synchronization errors as

$$\begin{array}{c} \phi_{e}=\int_{t_{0}}^{t_{1}}2\pi \;\left[f_{R}\left(t\right)-f_{T}\left(t\right)\right]\;\text{d}t+\left(\varphi_{d1}-\varphi_{d0}\right). \end{array}$$ (9)

Since

$$\begin{array}{c} \begin{array}{c} |\varphi_{R0}-\varphi_{T0}|=\left|\int_{t_{1}}^{t_{1}+t_{d}}2\pi f_{R}\left(t\right)\text{d}t-\int_{t_{0}}^{t_{0}+t_{d}}2\pi f_{R}\left(t\right)\text{d}t\right|\ll \frac{\pi }{4}, \end{array} \end{array}$$ (10)

(9) can be approximated as

$$\begin{array}{c} \phi_{e}=\int_{0}^{t_{1}-t_{0}}2\pi \;\left[f_{R}-f_{T}\right]\;\text{d}t. \end{array}$$ (11)

Since image generation with distributed radar requires a frequency coherence for at least one aperture time, namely *t*_1_ − *t*_0_ > *T*_*s*_ with *T*_*s*_ being the synthetic aperture time. For spaceborne radar systems, the typical synthetic aperture time is 1 seconds, while the typical synthetic aperture time is 5–15 seconds for airborne radar systems [32]. Moreover, phase synchronization errors are usually random and too complex to use autofocus image formation algorithms to obtain focused distributed radar image. Therefore, some synchronization compensation technique or compensation algorithms must be applied for distributed radar imaging.

### System Geometry and Transponder Arrangement

**Fig 1:**
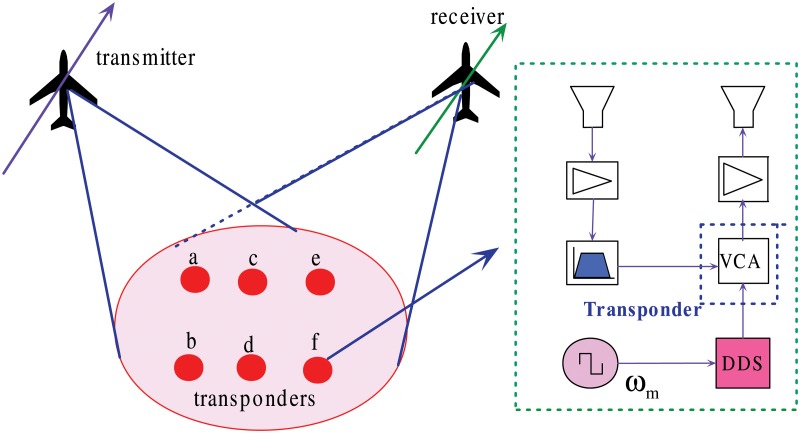
Transponder-aided joint calibration and synchronization system geometry.

Fig. 1 shows the system geometry of the transponder-aided joint radiometric calibration and frequency synchronization for distributed radar imaging, in which six transponders are placed in the observed scene. Each transponder consists of a low-noise amplifier followed by a band-pass filter. A voltage controlled attenuator (VCA) is used to modulate the radar signal in a manner that the retransmitted signal shows two additional Doppler shifts. Each transponder uses a distinct sinusoidal modulation frequency. The modulation frequency *ω*_*m*_ for the *m*th transponder is controlled by a direct digital synthesizer (DDS). Thereafter, the signal is amplified to an appropriate level and retransmitted towards the receiver. Note that two antennas are used in each transponder, one for transmitting and the other for receiving to minimize cross-coupling interferences.

Suppose the radar transmitted signal is a linearly frequency modulation (LFM) pulse signal

$$\begin{array}{c} s_{0}\left(t\right)=\exp\left\{j\omega_{t}t+j\pi k_{r}t^{2}\right\}, \end{array}$$ (12)

where *k*_*r*_ is the chirp rate and *ω*_*t*_ = 2*πf*_*t*_ with *f*_*t*_ being the transmitter carrier frequency. As the transponder can be seen as an amplitude modulator, the signal modulated by the *m*th transponder can be represented by

$$\begin{array}{c} s_{t_{m}}\left(t\right)=\left[\alpha_{m}+\beta_{m}\cos\left(\omega_{m}t+\varphi_{m}\right)\right]\;s_{m}\left(t\right), \end{array}$$ (13)

where *α*_*m*_ and *β*_*m*_ are constants determined by the *m*th transponder, *φ*_*m*_ is the starting phase and *s*_*m*_(*t*) is the signal arriving at the *m*th transponder. The *ω*_*m*_ and *φ*_*m*_ have direct relation to the receiver oscillator frequency, from which we can extract the phase synchronization errors (will be further investigated in Section 5). The signal coming to the distributed radar receiver is

$$\begin{array}{c} s_{a_{m}}\;\left(t\right)=s_{u_{m}}\;\left(t-\tau_{m}\right)+\left[\alpha_{m}+\beta_{m}\cos\left(\omega_{m}t+\varphi_{m}\right)\right]\;s_{m}\left(t-\tau_{m}\right), \end{array}$$ (14)

where *s*_*u*_*m*__(*t*) denotes the normal radar returns unmodulated by the transponder and *τ*_*m*_ is the time-delay required for the signal transmitting from the transmitter to the receiver.

Fourier transforming (14) with respect to *t* yields

$$\begin{array}{c} \begin{array}{cc} S_{a_{m}}\left(\omega \right)= & S_{u_{m}}\left(\omega \right)+\alpha_{m}e^{-j\omega \tau_{m}}S_{m}\;\left(\omega \right)+\frac{\beta_{m}}{2}e^{-j\varphi_{m}}S_{m}\;\left(\omega +\omega_{m}\right)+\frac{\beta_{m}}{2}e^{j\varphi_{m}}S_{m}\;\left(\omega -\omega_{m}\right), \end{array} \end{array}$$ (15)

where *S*_*m*_(*ω*) is the Fourier transforming of *s*_*m*_(*t* − *τ*_*m*_). Since *S*_*u*_*m*__(*ω*) ≐ *S*_*m*_(*ω*), the upper and lower side bands of the *m*th transponder signal are represented in frequency domain, respectively, by [28]

$$\begin{array}{c} s_{{\text{up}}_{m}}\left(t\right)=\frac{\beta_{m}}{2}e^{j\varphi_{m}}s_{m}\;\left(t-\tau_{m}\right)\;e^{j\omega_{m}t}, \end{array}$$ (16a)

$$\begin{array}{c} s_{{\text{dn}}_{m}}\left(t\right)=\frac{\beta }{2}e^{-j\varphi_{m}}s_{m}\;\left(t-\tau_{m}\right)\;e^{-j\omega_{m}t}. \end{array}$$ (16b)

We then have

$$\begin{array}{c} y_{m}\left(t\right)=\frac{s_{{\text{up}}_{m}}\left(t\right)}{s_{{\text{dn}}_{m}}\left(t\right)}=e^{j2[\omega_{m}t+\varphi_{m}]}. \end{array}$$ (17)

The estimates of *φ*_*m*_ and *ω*_*m*_ denoted as ${\hat{\varphi }}_{m}$ and ${\hat{\omega }}_{m}$ can then be used for subsequent motion and synchronization compensation.

### Transponder Signal Parameters Estimation

When noise is considered, (17) can be represented by a general signal model

$$\begin{array}{c} y_{m}\;\left(t\right)=a_{m}e^{j2[\omega_{m}t+\varphi_{m}]}+n_{m}\;\left(t\right), \end{array}$$ (18)

where *a*_*m*_ is the signal amplitude, and *n*_*m*_(*t*) is complex additive white Gaussian noise with zero mean and variance *σ* ^2^. Suppose the sampling interval is *T*_*s*_ and *N*_*s*_ samples of noisy discrete-time observations are available, we then have

$$\begin{array}{c} y_{m}\left[k\right]=a_{m}e^{j2[\omega_{m}kT_{s}+\varphi_{m}]}+n_{m}\left[k\right],\;k=0,1,\ldots ,N_{s}-1, \end{array}$$ (19)

where *n*_*m*_[*k*] = *n*_*m*_(*kT*_*s*_). We assume the modulo-2*π* reduced frequency *ω*_*m*_ has a probability density function (PDF) given by [33]

$$\begin{array}{c} p\left(\omega \right)=\frac{1}{2\pi },\;-\frac{\pi }{T_{s}}\leq \omega <\frac{\pi }{T_{s}}, \end{array}$$ (20)

and the modulo-2*π* reduced phase *φ*_*m*_ also has a PDF given by

$$\begin{array}{c} p\left(\varphi \right)=\frac{1}{2\pi },\;-\pi \leq \varphi <\pi . \end{array}$$ (21)

This statistical model is a specific Tikhonov distribution which has been widely used in modeling the statistics of frequency/phase estimation errors [34], and is of sufficient practical importance.

We want an estimate of *ω*_*m*_ and *φ*_*m*_ based on all the data samples *y*_*m*_[*k*], 0 ≤ *k* ≤ *N*_*s*_ − 1. The values of *ω*_*m*_ and *φ*_*m*_ are estimated by maximizing the PDF given by [33]

$$\begin{array}{c} p\;\left(\omega_{m},\varphi_{m}|{\left\{y_{m}\left[k\right]\right\}}_{k=0}^{N_{s}-1}\right)=\frac{p\;\left({\left\{y_{m}\left[k\right]\right\}}_{k=0}^{N_{s}-1}|\omega_{m},\varphi_{m}\right)\;p\left(\omega_{m},\varphi_{m}\right)}{p\;\left({\left\{y_{m}\left[k\right]\right\}}_{k=0}^{N_{s}-1}\right)}. \end{array}$$ (22)

Since $p\;\left({\left\{y_{m}[k]\right\}}_{k=0}^{N_{s}-1}\right)$ is independent of *ω*_*m*_ and *φ*_*m*_, maximizing (22) is equivalent to maximizing

$$\begin{array}{c} \begin{array}{cc} \wedge (\omega_{m},\varphi_{m})= & p\;\left({\left\{y_{m}\left[k\right]\right\}}_{k=0}^{N_{s}-1}|\omega_{m},\varphi_{m}\right)\;p\left(\omega_{m},\varphi_{m}\right)\\ = & \frac{C}{4\pi^{2}}\exp\left\{\frac{2a_{m}}{\sigma^{2}}\sum_{k=0}^{N_{s}-1}\left|y_{m}\left[k\right]\right|\cos\left[\text{arg}\left(y_{m}\left[k\right]\right)-\left(\omega_{m}k+\varphi_{m}\right)\right]\right\}, \end{array} \end{array}$$ (23)

where arg{*y*_*m*_[*k*]} denotes the measurement phase of *y*_*m*_[*k*] and *C* is

$$\begin{array}{c} C={\left(\frac{1}{\pi \sigma^{2}}\right)}^{N_{s}}\exp\left\{-\sum_{k=0}^{N_{s}-1}\frac{\left(|y_{m}\left[k\right]{|}^{2}+a_{m}^{2}\right)}{\sigma^{2}}\right\}. \end{array}$$ (24)

Taking the natural logarithm of both sides yields

$$\begin{array}{c} \log\wedge \left(\omega_{m},\varphi_{m}\right)=\log\left(\frac{C}{4\pi^{2}}\right)+\frac{2a_{m}}{\sigma^{2}}\;\sum_{k=0}^{N_{s}-1}\left|y_{m}\left[k\right]\right|\cos\left[\text{arg}\left(y_{m}\left[k\right]\right)-\left(\omega_{m}k+\varphi_{m}\right)\right]. \end{array}$$ (25)

Suppose *ω*_*m*_ and *φ*_*m*_ are statistically independent of each other, the maximum likelihood estimates ${\hat{\omega }}_{m}^{\left(N_{s}-1\right)}$ and ${\hat{\varphi }}_{m}^{\left(N_{s}-1\right)}$ of *ω*_*m*_ and *φ*_*m*_ can be computed, respectively, by [33]

$$\begin{array}{c} {\hat{\omega }}_{m}^{\left(N_{s}-1\right)}=\frac{\left[\sum_{k=0}^{N_{s}-1}k\left|y_{m}\left[k\right]\right|\text{arg}\left\{y_{m}\left[k\right]\right\}\right]\;\left[\sum_{k=0}^{N_{s}-1}\left|y_{m}\left[k\right]\right|\right]\;-\;\left[\sum_{k=0}^{N_{s}-1}\left|y_{m}\left[k\right]\right|\text{arg}\left\{y_{m}\left[k\right]\right\}\right]\;\left[\sum_{k=0}^{N_{s}-1}k\left|y_{m}\left[k\right]\right|\right]}{2T_{s}\;\left\{\left[\sum_{k=0}^{N_{s}-1}k^{2}\left|y_{m}\left[k\right]\right|\right]\;\left[\sum_{k=0}^{N_{s}-1}\left|y_{m}\left[k\right]\right|\right]\;-\;{\left[\sum_{k=0}^{N_{s}-1}k\left|y_{m}\left[k\right]\right|\right]}^{2}\right\}}, \end{array}$$ (26)

$$\begin{array}{c} {\hat{\varphi }}_{m}^{\left(N_{s}-1\right)}=\frac{\left[\sum_{k=0}^{N_{s}-1}k\left|y_{m}\left[k\right]\right|\text{arg}\left\{y_{m}\left[k\right]\right\}\right]\;\left[\sum_{k=0}^{N_{s}-1}k\left|y_{m}\left[k\right]\right|\right]\;-\;\left[\sum_{k=0}^{N_{s}-1}\left|y_{m}\left[k\right]\right|\text{arg}\left\{y_{m}\left[k\right]\right\}\right]\;\left[\sum_{k=0}^{N_{s}-1}k^{2}\left|y_{m}\left[k\right]\right|\right]}{2{\left[\sum_{k=0}^{N_{s}-1}k\left|y_{m}\left[k\right]\right|\right]}^{2}-2\;\left[\sum_{k=0}^{N_{s}-1}k^{2}\left|y_{m}\left[k\right]\right|\right]\;\left[\sum_{k=0}^{N_{s}-1}\left|y_{m}\left[k\right]\right|\right]}. \end{array}$$ (27)

It can be noticed that this estimation algorithm makes use of both the measurement phase arg{*y*_*m*_[*k*]} and measurement magnitude ∣*y*_*m*_[*k*]∣ of the received signal samples *y*_*m*_[*k*]; however, it requires neither the knowledge of the amplitude *a*_*m*_ nor that of the noise power *σ* ^2^.

Since the measurement phase arg{*y*_*m*_[*k*]} is obtained from the principal argument of the complex phasor *y*_*m*_[*k*], phase unwrapping is necessary. Various phase unwrapping algorithms have been proposed in the literature [35–38]. Considering the continuous updating nature of the phase synchronization error estimation, here we use the Kalman filter-based phase unwrapping algorithm proposed in [33]. This algorithm assume that at time point *k* the ML estimates ${\hat{\omega }}_{m}^{\left(k\right)}[k]$ and ${\hat{\varphi }}_{m}^{\left(k\right)}$ are computed from (26) and (27), respectively, and take ${\hat{\omega }}_{m}^{\left(k\right)}[k+1]$ and ${\hat{\varphi }}_{m}^{\left(k\right)}$ as the prediction of the next value for the estimated signal given measurements up to time *k*. Then the unwrapped phase is the one lying in the interval [33]

$$\begin{array}{c} \left[{\hat{\omega }}_{m}^{\left(k\right)}\;\left[k+1\right]T_{s}+{\hat{\varphi }}_{m}^{\left(k\right)}-\pi ,\;{\hat{\omega }}_{m}^{\left(k+1\right)}\;\left[k+1\right]T_{s}+{\hat{\varphi }}_{m}^{\left(k\right)}+\pi \right). \end{array}$$ (28)

This algorithm can be recursively implemented like the Kalman filter [39].

### Radiometric Calibration

According to (14), each transponder will yield two artificial Doppler signals in the distributed radar returns. Therefore, the distributed radar received signal corresponding to the *m*th transponder can be reexpressed as

$$\begin{array}{c} s_{r_{m}}\left(t\right)=\sigma_{0}\;s_{u_{m}}\left(t-\tau_{m}\right)+\frac{\beta_{m}\sigma_{0}}{2}e^{-j(\omega_{m}t+\varphi_{m})}\;s_{u_{m}}\left(t-\tau_{m}\right)+\frac{\beta_{m}\sigma_{0}}{2}e^{j(\omega_{m}t+\varphi_{m})}\;s_{u_{m}}\left(t-\tau_{m}\right). \end{array}$$ (29)

After being processed by general image formation algorithms, e.g., Range-Doppler imaging algorithms [40], the focused imagery will be [4]

$$\begin{array}{c} z_{m}\;\left(t,\tau_{m}\right)=\sigma_{0}\text{sinc}\;\left(t-t_{0}\right)\;\text{sinc}\;\left(\tau -\tau_{0}\right)+\frac{\beta_{m}\sigma_{0}}{2}\text{sinc}\;\left(t-t_{m}\right)\;\text{sinc}\;\left(\tau -\tau_{m_{1}}\right)+\frac{\beta_{m}\sigma_{0}}{2}\;\text{sinc}\;\left(t-t_{m}\right)\;\text{sinc}\;\left(\tau -\tau_{m_{2}}\right), \end{array}$$ (30)

where *t*_0_ and *τ*_0_ denote the fast-time and slow-time in the normal radar imagery, respectively, *t*_*m*_ is the fast-time for the transponder, *τ*_*m*_1__ and *τ*_*m*_2__ denote the slow-times for the two Doppler signals generated by the transponder. That is to say, each transponder will produce two artificial point-targets in the imagery. The transponder imagery can be quantitatively measured as *γ*_*m*_, namely

$$\begin{array}{c} \gamma_{m}=\frac{\beta_{m}\sigma_{0}}{2}. \end{array}$$ (31)

Since *β*_*m*_ is a known variable because it is measurable from the transponder signals, the quantitative radiometric coefficient *σ*_0_ can then be determined as

$$\begin{array}{c} \sigma_{0}=\frac{2\gamma_{m}}{\beta_{m}}. \end{array}$$ (32)

Since the transponder signal and SAR signal have similar channel effects (because the transponders are placed in the SAR imaging scene) and the transponder received and re-transmitted signal amplitudes can be measured in real-time on ground, the quantitative radiometric coefficient *σ*_0_ can then be determined by comparing the transponder received and re-transmitted signal amplitudes with SAR received signal amplitude. In doing so, the distributed radar imagery is effectively calibrated and thus we can measure the fading effect, which is an important phenomena for a coherent system [41].

As the feasibility of the transponder in radiation calibration has been fully investigated and validated in [27], this paper mainly discusses the aspects of motion compensation and frequency synchronization compensation in the transponder-aided joint calibration and synchronization compensation for distributed radar systems.

### Motion Compensation

In distributed radar imaging, as a matter of fact, motion compensation problems may arise due to the presence of atmospheric turbulence, which introduce platform trajectory deviations from nominal position, as well as altitude [42]. To account for such errors, onboard GPS and inertial navigation units are widely employed. If high-precision motion measurement sensors are not available, signal processing-based motion compensation must be applied.

The positions of the transmitter and receiver can be determined from the different pseudo-ranges and the knowledge of the transponder position (*x*_*m*_, *y*_*m*_, *z*_*m*_) as follows:

$$\begin{array}{cc} & \sqrt{{\left(x_{t}-x_{\text{1}}\right)}^{2}+{\left(y_{t}-y_{\text{1}}\right)}^{2}+{\left(z_{t}-z_{\text{1}}\right)}^{2}}+\sqrt{{\left(x_{r}-x_{\text{1}}\right)}^{2}+{\left(y_{r}-y_{\text{1}}\right)}^{2}+{\left(z_{r}-z_{\text{1}}\right)}^{2}}=R_{1}, \end{array}$$ (33a)

$$\begin{array}{cc} & \sqrt{{\left(x_{t}-x_{\text{2}}\right)}^{2}+{\left(y_{t}-y_{\text{2}}\right)}^{2}+{\left(z_{t}-z_{\text{2}}\right)}^{2}}+\sqrt{{\left(x_{r}-x_{\text{2}}\right)}^{2}+{\left(y_{r}-y_{\text{2}}\right)}^{2}+{\left(z_{r}-z_{\text{2}}\right)}^{2}}=R_{2}, \end{array}$$ (33b)

$$\begin{array}{cc} & \sqrt{{\left(x_{t}-x_{\text{3}}\right)}^{2}+{\left(y_{t}-y_{\text{3}}\right)}^{2}+{\left(z_{t}-z_{\text{3}}\right)}^{2}}+\sqrt{{\left(x_{r}-x_{\text{3}}\right)}^{2}+{\left(y_{r}-y_{\text{3}}\right)}^{2}+{\left(z_{r}-z_{\text{3}}\right)}^{2}}=R_{3}, \end{array}$$ (33c)

$$\begin{array}{cc} & \sqrt{{\left(x_{t}-x_{\text{4}}\right)}^{2}+{\left(y_{t}-y_{\text{4}}\right)}^{2}+{\left(z_{t}-z_{\text{4}}\right)}^{2}}+\sqrt{{\left(x_{r}-x_{\text{4}}\right)}^{2}+{\left(y_{r}-y_{\text{4}}\right)}^{2}+{\left(z_{r}-z_{\text{4}}\right)}^{2}}=R_{4}, \end{array}$$ (33d)

$$\begin{array}{cc} & \sqrt{{\left(x_{t}-x_{\text{5}}\right)}^{2}+{\left(y_{t}-y_{\text{5}}\right)}^{2}+{\left(z_{t}-z_{\text{5}}\right)}^{2}}+\sqrt{{\left(x_{r}-x_{\text{5}}\right)}^{2}+{\left(y_{r}-y_{\text{5}}\right)}^{2}+{\left(z_{r}-z_{\text{5}}\right)}^{2}}=R_{5}, \end{array}$$ (33e)

$$\begin{array}{cc} & \sqrt{{\left(x_{t}-x_{\text{6}}\right)}^{2}+{\left(y_{t}-y_{\text{6}}\right)}^{2}+{\left(z_{t}-z_{\text{6}}\right)}^{2}}+\sqrt{{\left(x_{r}-x_{\text{6}}\right)}^{2}+{\left(y_{r}-y_{\text{6}}\right)}^{2}+{\left(z_{r}-z_{\text{6}}\right)}^{2}}=R_{6}. \end{array}$$ (33f)

There are six unknown parameters, namely, (*x*_*t*_, *y*_*t*_, *z*_*t*_) and (*x*_*r*_, *y*_*r*_, *z*_*r*_), and six independent equations. Certainly, if a basic ranging method is employed, we cannot achieve the position at a fractional of the wavelength. To overcome this problem, in this paper the transponder position is obtained by taking each transponder as a specific target and comparing the relative distance between each transponder imagery and normal radar imagery with the method similar to that the strong target based inverse radar autofocus technique. Thus, all the unknown parameters are solely determinable. These position information can then be used for compensating the motion errors in subsequent distributed radar image formation processing.

### Frequency Synchronization Compensation

The transponder signal can also be used for compensating the frequency synchronization errors. As the synchronization errors can be extracted by one transponder, in the following we consider only one transponder.

Similar to existing literatures, physical frequency is used in section to discuss the frequency synchronization problem. To find a general mathematical model, suppose the *n*th transmitted pulse is

$$\begin{array}{c} s_{\text{tn}}\;\left(t\right)=\text{rect}\;\left[\frac{t}{T_{p}}\right]\exp\left[j\left(2\pi f_{\text{tn}}t+k_{r}t^{2}+\varphi_{e}\left(n\right)\right)\right], \end{array}$$ (34)

where rect[⋅] is the window function, *f*_tn_ is the transmitter carrier frequency, *k*_*r*_ is the chirp rate, *T*_*p*_ is the pulse duration and *φ*_*e*_(*n*) is the original phase to be estimated. Suppose the demodulating signal in receiver is

$$\begin{array}{c} s_{\text{rn}}\;\left(t\right)=\exp\left(j2\pi f_{\text{rn}}t\right). \end{array}$$ (35)

Hence the received transponder signal (can be seen as a strong point target) in baseband is

$$\begin{array}{c} \begin{array}{cc} s_{\text{bn}}\;\left(t\right)= & \text{rect}\;\left[\frac{t-\tau_{n}}{T_{p}}\right]\;\cdot \text{exp}\left(j\varphi_{e}\left(n\right)\right)\;\text{exp}\left[j2\pi \left(f_{\text{tn}}-f_{\text{rn}}\right)t\right]\;\cdot \text{exp}\left[-j2\pi \left(f_{\text{tn}}+f_{\text{dn}}\right)\tau_{n}\right], \end{array} \end{array}$$ (36)

where *f*_rn_ is the the receiver carrier frequency, *f*_dn_ is the Doppler shift, and *τ*_*n*_ is the time delay corresponding to the time it takes the signal to travel the transmitter-transponder-receiver distance for the *n*th pulse.

Let Δ*f*_*n*_ = *f*_tn_ − *f*_rn_, and suppose the range reference function is

$$\begin{array}{c} s_{\text{cn}}\;\left(t\right)=\text{rect}\;\left[\frac{t}{T_{p}}\right]\;\text{exp}\left(-j2\pi k_{r}t^{2}\right). \end{array}$$ (37)

Range compressing yields

$$\begin{array}{c} \begin{array}{cc} s_{on}\;\left(t\right)= & \text{sinc}\;\left[\left(k_{r}T_{p}-\Delta f_{n}\right)\;\left(t-\tau_{n}-\frac{\Delta f_{n}}{k_{r}}\right)\right]\;\left(k_{r}T_{p}-\Delta f_{n}\right)\;\cdot \text{exp}\;\left[j\pi \Delta f_{n}\left(t-\tau_{n}+\frac{\Delta f_{n}}{k_{r}}\right)\right]\\ & \times \exp\left\{-j2\pi \left(f_{dn}+f_{rn}\right)\tau_{n}+j\pi \frac{{\Delta f_{n}}^{2}}{k_{r}}+j\varphi_{e}\left(n\right)\right\}. \end{array} \end{array}$$ (38)

We can notice that the maxima will be at $t=t_{\text{dn}}-\frac{\Delta f_{n}}{k_{r}}$ when

$$\begin{array}{c} \text{exp}[j\pi \Delta f_{n}\left(t-\tau_{n}+\Delta f_{n}/k_{r}\right]{|}_{t=\tau_{n}-\Delta f_{n}/k_{r}}=1. \end{array}$$ (39)

Then the residual phase term in (38) is

$$\begin{array}{c} \psi \left(n\right)=-2\pi \left(f_{\text{dn}}+f_{\text{rn}}\right)\tau_{n}-\frac{\pi {\Delta f_{n}}^{2}}{k_{r}}+\varphi_{e}\left(n\right). \end{array}$$ (40)

As Δ*f*_*n*_ and *k*_*r*_ are typically on the orders of 1 kHz and 1 × 10^13^ Hz/s, respectively, $\pi {\Delta f_{n}}^{2}/k_{r}$ has negligible effects.

If let

$$\begin{array}{c} f_{\text{rn}}=f_{\text{r0}}+\delta f_{\text{rn}}, \end{array}$$ (41a)

$$\begin{array}{c} f_{\text{dn}}=f_{\text{d0}}+\delta f_{\text{dn}}, \end{array}$$ (41b)

where *f*_d0_ and *f*_r0_ are the original Doppler shift and error-free demodulating frequency in receiver, respectively. Accordingly, *δf*_dn_ and *δf*_rn_ are the frequency errors for the *n*th pulse. Hence we have

$$\begin{array}{c} \begin{array}{cc} \varphi_{e}\left(n+1\right)-\varphi_{e}\left(n\right)= & \left[\psi \left(n+1\right)-\psi \left(n\right)\right]-2\pi \left(f_{\text{r0}}+f_{\text{d0}}\right)\;\left(\tau_{n+1}-\tau_{n}\right)-2\pi \left(\delta f_{\text{dn}}+\delta f_{\text{rn}}\right)\;\left(\tau_{\text{n+1}}-\tau_{n}\right). \end{array} \end{array}$$ (42)

Generally speaking, *δf*_dn_ + *δf*_rn_ and *τ*_n + 1_ − *τ*_*n*_ are typically on the orders of 10 Hz and 10^−9^ sec., respectively, then 2*π*(*δf*_dn_ + *δf*_rn_)(*τ*_n + 1_ − *τ*_*n*_) is found to be smaller than 2*π* × 10^−8^ radian, which has negligible effects. Thus, (42) can be further simplified into

$$\begin{array}{c} \begin{array}{cc} \varphi_{e}\left(n+1\right)-\varphi_{e}\left(n\right)= & \left[\psi \left(n+1\right)-\psi \left(n\right)\right]-2\pi \left(f_{\text{r0}}+f_{\text{d0}}\right)\;\left(\tau_{\text{n+1}}-\tau_{n}\right). \end{array} \end{array}$$ (43)

At this step, the estimation of the synchronization errors *φ*_*e*_(*n*) can be calculated according to (43).

In conventional synchronization links, the transmitter must transmit an additional synchronization signal to the receiver. Moreover, the synchronization signal must be sufficiently decoupled from the normal radar signal, so that they can effectively separated in subsequent synchronization compensation algorithm. Different from conventional methods, the proposed approach requires neither the transmitter transmits an additional synchronization signal to the receiver, nor a duplex hardware system. Thus, when compared to conventional synchronization links, the proposed synchronization approach has a lower hardware system complexity.

Moreover, the synchronization signal in conventional synchronization methods may be impacted by the normal radar signal or bring interferences on the radar signal. But the transponder-based approach is not impacted by the normal radar signal. If the designed transponder modulation frequency *ω*_*m*_ is larger than the Doppler shift

$$\begin{array}{c} \omega_{m}=2\pi f_{m}\geq \frac{2\pi v_{a}}{\lambda }, \end{array}$$ (44)

where *v*_*a*_ is the relative velocity between the radar platform and the observed target. The transponder signal can be easily filtered out at the receiver through (15)–(16).

## Results

### Simulation Parameters

In all the simulations, we suppose the transmitted radar signal is a LFM signal with the following parameters: carrier frequency is *f*_*c*_ = 1.2 GHz, bandwidth is *B* = 50 MHz, pulse duration is *T*_*p*_ = 5*μs* and PRF is 1500 Hz.

### Transponder Modulation Results

**Fig 2:**
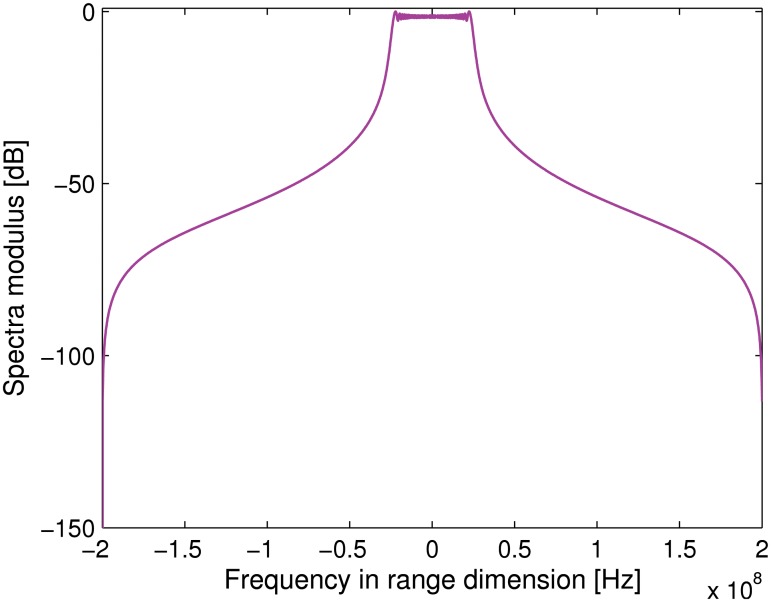
Spectra transmitted by the distributed radar transmitter.

**Fig 3:**
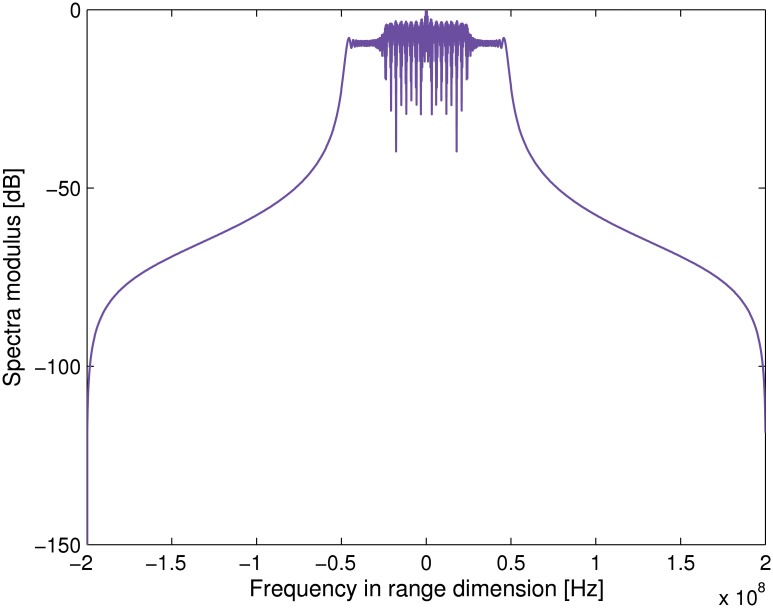
Transponder received signal spectra.

**Fig 4:**
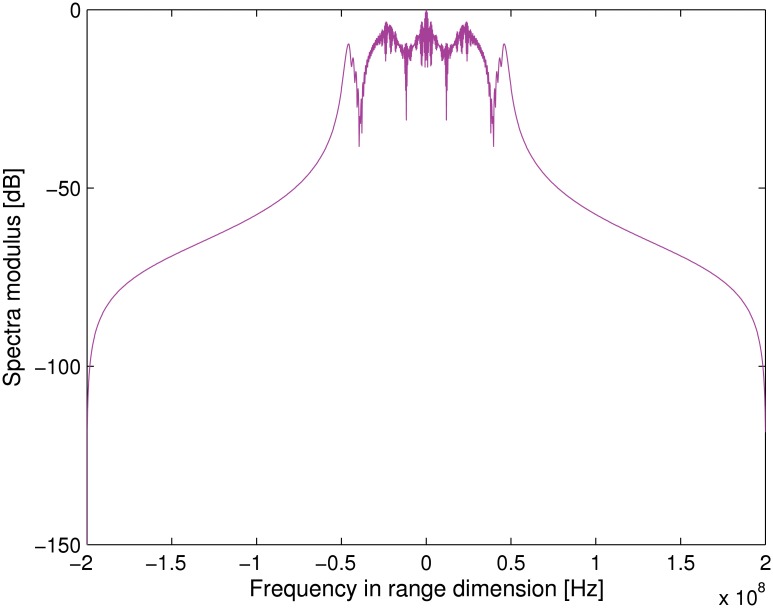
Transponder retransmitted signal spectra.

The corresponding transmitted signal spectrum is shown in Fig. 2. Note that the carrier frequency is shifted to the zero frequency. We further assume there are three point targets with the slant range of −700 m, 0 m and 700 m, respectively. Figs. 3 and 4 compares the normalized spectra of the signal received and transmitted by the transponder. Note that *f*_*m*_ = 500 Hz is assumed in the simulation. It can be noticed that, after modulated by the transponder, there is only a small bandwidth extension when compared to the unmodulated radar signal. Therefore, it is not necessary to make any hardware modification to radar receiver. methods.

Next, after matched filtering and range pulse compressing in a manner like the general radar signal processing, the transponder signal can be easily extracted due to the amplitude modulation which results in a small ‘‘Doppler’’ shift. The phase synchronization errors can then be estimated by comparing the frequency distance between the original azimuth signal and the additional frequency shifted signals.

### Calibration Errors Estimation Results

**Fig 5:**
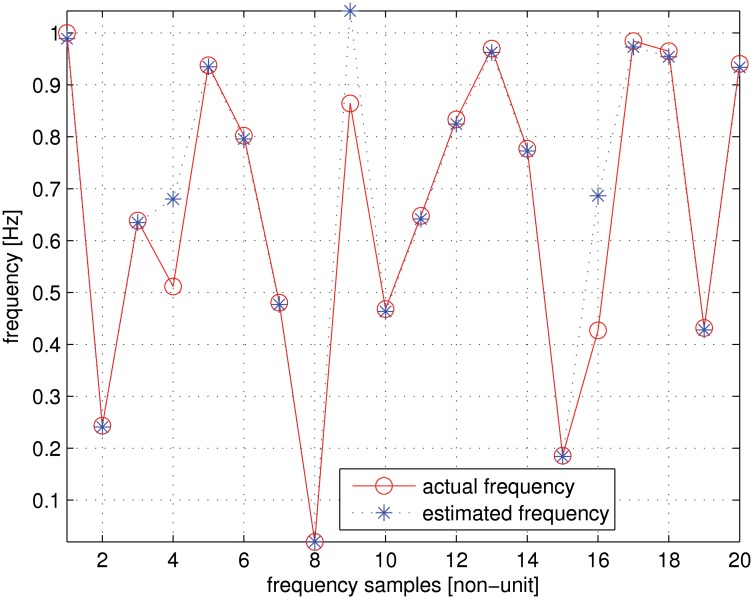
Comparative frequency estimation results.

We suppose the transponder signal is *y*_*m*_(*t*) = 5*e* ^*j*(*ω*_*m*_*t*+*θ*_*m*_)^+2.5*n*(*t*) (see (18)), where *n*(*t*) is the Gaussian distributed noise with mean zero and unit covariance. Note that the normalized *ω*_*m*_ is used in the simulation. Using the analytical oscillator frequency instability model that we developed in [43], we simulated the possible frequency synchronization errors. Fig. 5 shows the estimate performance of the maximum likelihood estimator. Note that the frequency synchronization errors shown in Fig. 5 have been normalized and *θ*_*m*_ = 0 is assumed in this case. It can be noticed that satisfactory performance is achieved for the estimator.

**Fig 6:**
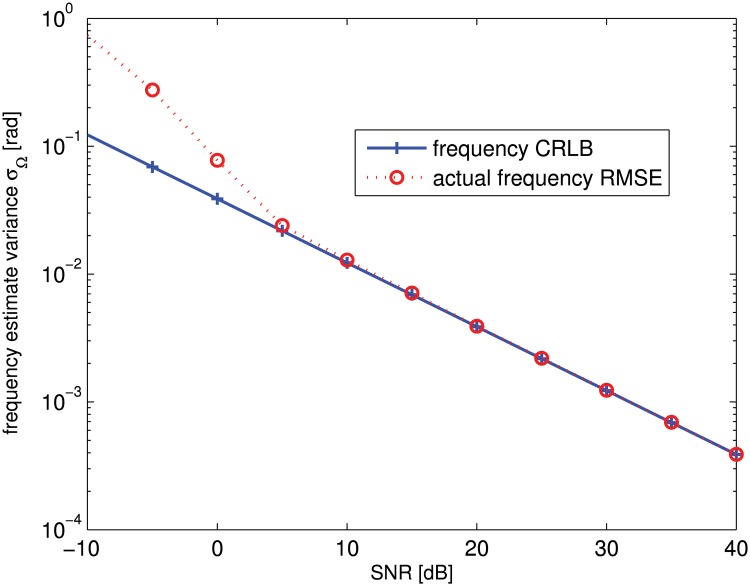
Frequency estimate variance versus the SNR parameter.

Although wideband radar signal is used in the simulation example, the signal that we use to estimate the carrier synchronization errors is a narrow-band signal because the transponder modulation signal is a monochromatic signal. Fig. 6 shows the cramér lower bound (CRLB) and root-mean-square errors (RMSE) on the frequency estimate versus signal-to-noise ratio (SNR) parameter. Note that the estimation RMSE are computed based on 500 independent runs. It can be seen that the estimator gives a satisfactory estimation performance.

### Synchronization Compensation Results

**Fig 7:**
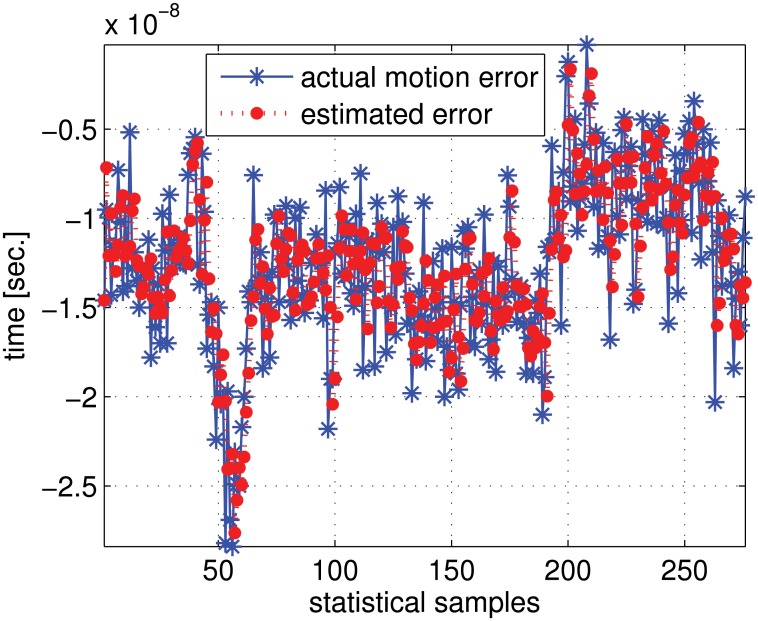
Comparative motion errors and our estimated motion errors.

To evaluate the motion compensation method, we performed statistically simulation investigations using the actual GPS data obtained from the IGS website (http://igscb.jpl.nasa.gov/). Fig. 7 compares the actual motion errors and our estimated motion errors. From the positive results we can conclude that the motion errors can be compensated by jointly exploiting the six transponder signals.

**Fig 8:**
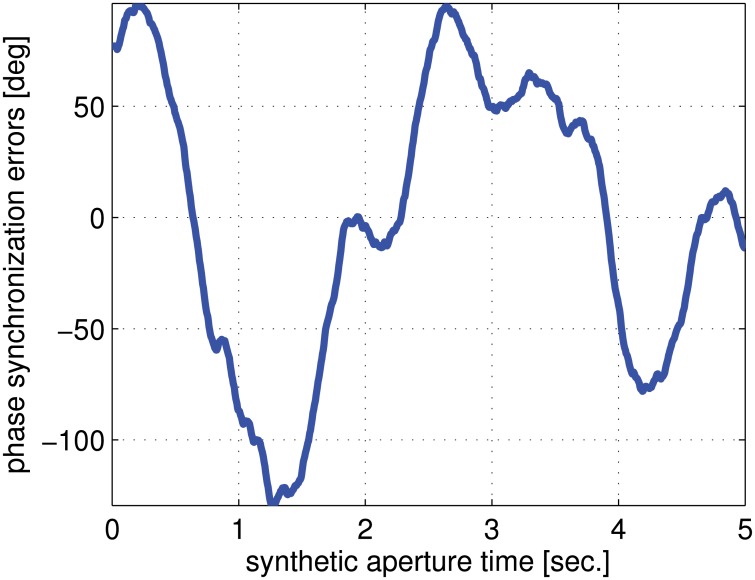
Phase synchronization errors assumed in phase synchronization simulation.

**Fig 9:**
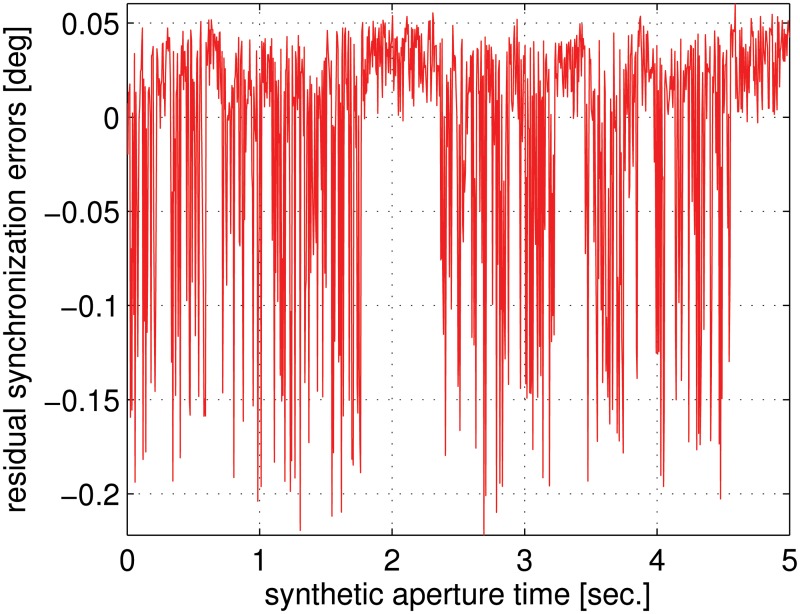
Residual phase synchronization errors after applying the transponder-aided compensation method.

Finally, to evaluate the performance of this transponder-aided phase synchronization method, we consider a bistatic synthetic aperture radar system with the phase synchronization errors shown in Fig. 8. Using the proposed phase synchronization method, Fig. 9 shows the residual phase synchronization errors. It can be noticed that the residual phase synchronization errors fall into −0.2–−0.05 degree, which have ignorable impacts on most of distributed radar systems. This implies that phase synchronization errors can be effectively compensated by the transponder-aided estimation and compensation method.

## Discussion

High-precision radiometric calibration, motion and synchronization compensation must be ensured for distributed radar, which operates with separate transmitters and receivers. This article proposes a joint radiometric calibration and synchronization compensation based on the transponders for distributed radar imaging. This method requires no hardware modifications in both the normal radar transmitter and receiver (the PRF is also not changed). It also does not change the range ambiguity characteristics of the normal radar. All the proposed methods are verified by simulation results. Although a high accuracy of the synchronization and motion compensation can be obtained if there is a transponder in each acquired image, it is not necessary because we can use the transponder to synchronize and calibrate the radar system intermittently at a time interval and compensate the remaining synchronization errors with autofocus processing algorithms. Another note is that only one transponder is required for bistatic radar frequency synchronization compensation. However, since at least variables are required to locate the bistatic radar transmitter and receiver, 6 transponders are needed to form six independent equations, so that their relative positions can be determined, equivalently the motion errors can be compensated.
